# HIV and Aging Comes into the Spotlight: 2013 to Now

**DOI:** 10.1093/geroni/igaa057.3114

**Published:** 2020-12-16

**Authors:** Tonya Taylor

**Affiliations:** SUNY Downstate Health Sciences University, Brooklyn, New York, United States

## Abstract

I have had the pleasure of serving as Co-Convener of the HIV, AIDS and Older Adults Special Interest Group (SIG) for seven years (2013 to now). During this time, we witnessed an increase in NIH funding opportunities, most notable was the Multidisciplinary Studies in HIV/AIDS and Aging FOA. NIH also renewed its support for two prominent longitudinal HIV cohort studies (now knowns as the Combined Cohort Study) because of their unmatched ability to provide insight on the effects of HIV infection and aging. Individuals older than 50 years of age are now included in AIDS and HIV prevention clinical trials. And, in 2008 the AIDS Institute launched the first National HIV/AIDS and Aging Awareness Day (Sept 18th). During this period, the SIG continued to raise awareness about HIV and aging through sponsored symposia and Webinars, and participants for the SIG participated in the first HIV and aging GSA Momentum Discussion. Part of a symposium sponsored by the HIV, AIDS and Older Adults Interest Group.

